# Common Genetic Variant of *INSIG2* Gene rs7566605 Polymorphism Is Associated with Severe Obesity in North India

**DOI:** 10.18869/acadpub.ibj.21.4.261

**Published:** 2017-07

**Authors:** Jai Prakash, Balraj Mittal, Apurva Srivastava, Shally Awasthi, Pranjal Srivastava, Neena Srivastava

**Affiliations:** 1 Department of Physiology, King George’s Medical University, Lucknow, Uttar Pradesh, India; 2 Department of Pediatrics, King George’s Medical University, Lucknow, Uttar Pradesh, India; 3 Department of Genetics, Sanjay Gandhi Postgraduate Institute of Medical Sciences, Lucknow, U.P., India; 4 Darbhanga Medical College and Hospital Near Karpuri Chowk Benta Laheriasarai Darbhanga Bihar 846003, India

**Keywords:** INSIG2, Body mass index, Insulin, Insulin resistance, Polymorphism

## Abstract

**Background:**

Obesity is a very common disorder resulting from an imbalance between food intake and energy expenditure, and it has a substantial impact on the development of chronic diseases. The aim of this study was to examine the association of *INSIG2* (rs7566605) gene polymorphism with obesity and obesity associated phenotypes in North Indian subjects.

**Methods:**

The variants were investigated for association in 642 obese and non-obese individuals. The genotyping of *INSIG2* (rs7566605) single nucleotide polymorphism was analyzed by the TaqMan allelic discrimination protocol.

**Results:**

A significant association was observed for *INSIG2* (rs7566605) single nucleotide polymorphism with obesity and obesity-related phenotypes. Furthermore, a significant relationship was found between the rs7566605 and insulin, homeostasis model of assessment-insulin resistance, the percentage of body fat, fat mass, leptin, and adiponectin.

**Conclusion:**

The present study observed significant association between *INSIG2* (rs7566605) single nucleotide polymorphism and obesity, as well as obesity-associated phenotypes in North Indian population.

## INTRODUCTION

Obesity results from an disparity among food intake and energy consumption and is a major risk factor for many chronic diseases of adulthood such as type 2 diabetes and coronary heart disease[1,2]. Although recent estimates have reported that >12% of the entire population is overweight or obese[3], the results of twin and adoption studies have strongly supported the impact of genetics on the variation of obesity[4,5], as well as the heritability of body mass index (BMI)[6-8].

In a genome-wide association study, Herbert and coworkes[9] identified a single nucleotide polymorphism (SNP) (rs7566605) upstream of the insulin-induced gene 2 *(INSIG2)* and observed a significant association between SNP (rs7566605) and increased BMI. On the other hand, Talbert *et al*.[10] found a correlation in some of the *INSIG2* variants, but not with rs7566605, with the adiposity and biochemical indicators of glucose homeostasis. An association between rs7566605 and obesity has also been reported in different ethnic groups[9,11-13], though these associations have not supported by some studies [14-16]. Furthermore, no relationship was observed in the Indian population[17,18]. Thus, the association between rs7566605 in the *INSIG2* gene and obesity remains controversial.

The SNP rs7566605 is located at 10-kb upstream of the transcription start site of the *insig2* and has no known function. *INSIG1* encodes an endoplasmic reticulum membrane protein that regulates cholesterol[19] and glucose homeostasis[20]. *INSIG2* is a ~21.5-kb gene located on chromosome 2q14 and encodes INSIG2 protein, which has been functionally related to lipid metabolism by the inhibition of cholesterol and fatty acid synthesis[21,22].

Sterol response element-binding protein (SREBP) is an endoplasmic reticulum membrane bound protein that prevents the proteolytic activation of another protein, in response to cholesterol or insulin[21]. This protein resides in the endoplasmic reticulum where it binds to SREBP cleavage-activating protein to inhibit it from convoying SREBPs to the Golgi apparatus[22]. Ultimately, *INSIG2* inhibits SREBP from stimulating cholesterol synthesis as SREBP cannot be treated and triggered by the Golgi enzymes. These functional effects of *INSIG2* gene have also been observed in mice[22,23]. Animal data signifies the role of *INSIG2* in the regulation of cholesterol in rats[24]. However, such observations have not been established in human populations[15,18,25,26].

The prevalence of hypercholesterolemia has been shown that to be associated with CC homozygotes of rs7566605; however, the SNP rs7566605 was not related to the levels of total cholesterol, high density lipoprotein cholesterol (HDL-C), or triacylglycerol[27]. Data mapping of quantitative trait loci in mice for obesity and obesity-related traits and their response to high-fat diet have shown the linkage of the *INSIG2* gene with fat depots and serum cholesterol levels[28].

To confirm the above reported findings, we examined the association of rs7566605 polymorphism with obesity defined by BMI and obesity-associated phenotypes; particularly with systolic blood pressure, diastolic blood pressure, serum concentrations of glucose, insulin, and with a surrogate measure of insulin resistance, i.e. the homeostasis model assessment (HOMA) index of insulin resistance. The percentage of body fat, fat mass, total cholesterol, high density lipoprotein cholesterol (HDL-C), low density lipoprotein cholesterol (LDL-C), very low density lipoprotein cholesterol, triglyceride, leptin, and adiponectin were also examined in obese and non-obese individuals from Lucknow, India.

## MATERIALS AND METHODS

### Patients

All individuals participated in the present study were of North Indian origin and belonged to the states of Delhi, Haryana, Jammu and Kashmir, Himachal Pradesh, Uttar Pradesh, Punjab, and Uttarakhand, and those who were not of North Indian origin were excluded. The population was homogeneous with regard to ethnic background, as described in our previous study[29]. Prior to the experiments, an informed written consent was taken from each participant, and the identity of all the participants was kept confidential. The study was carried out with the approval of local ethics committee at King George’s Medical University Lucknow, Uttar Pradesh, India, and the study protocol was conformed to the ethical guidelines of the 1975 Declaration of Helsinki.

All participants were subjected to a careful screening program that included the assessment of a detailed personal and family history, physical examination, determination of anthropometric measurements, and biochemical profiles. BMI was measured for all the participants. After screening of 821 subjects, we selected 642 cases on the basis of strict inclusion/exclusion criteria.

### Inclusion and exclusion criteria for obese and non-obese subjects

The inclusion criteria for the subjects were having North India origin, having the age of 20-42 years at time of interview, as well as BMI≥30 kg/m^2^ for obese and BMI 18.5-29.99 kg/m^2^ for non-obese cases. Subjects who did not fulfil the inclusion criteria and those with congenital and mental disorders, as well as endocrine disorders such as myxoedema, Cushing’s syndrome and metabolic disorders like diabetes mellitus, cardiovascular disease, congestive heart failure, and pregnant women were excluded. In total, 309 obese subjects (BMI≥30) and 333 non-obese cases (BMI<30) were enrolled in the study. The participants were selected from general population in Lucknow City (Uttar Pradesh, India).

### Biochemical parameters

Blood samples (5 ml) were collected from obese and non-obese individuals after an overnight fasting. Within one hour of collection, the samples were centrifuged at room temperature for three min to obtain plasma/serum, frozen in aliquots and then stored at -80°C until analysis. Commercial enzymatic test kits (ERBA Diagnostics Mannheim GmbH, Germany) were used to determine HDL-C, triglyceride concentrations, and total cholesterol. LDL-C was calculated by the Friedewald formula (LDL-C=total cholesterol-HDL-C-triglyceride/5 mg/dL). The inter-assay coefficient of variation was <5.0% for HDL-C and <2.5% for triglycerides[30]. Adiponectin was assayed with enzyme-linked immunosorbent assay method. Insulin and leptin levels were determined by enzyme-linked radio immunosorbent assay (Linco Research Inc., USA). The inter-assay coefficient of variation for the insulin assay was 5.7%[31]. The intra-assay coefficient of variation for leptin was 3.4 to 8.3%, and the inter-assay coefficient of variation was 3.0 to 6.2%. Samples were assayed in duplicate, and the limits of detection and linearity for the leptin radioimmunoassay were 0.5 and 100 ng/ml[32].

The degree of insulin sensitivity/resistance was calculated according to the HOMA, which is a good index for assessing insulin sensitivity/resistance. Based on HOMA, insulin resistance was calculated as described previously[33]. The fasting glucose concentration was measured by glucose oxidase-peroxidase method[34]. A body fat analyzer (Tanita–TBF–310, Japan) was used to assess the percentage of body fat (fat mass). The body fat analyzer had previously been validated based on bioelectrical impedance[35].

### Genotyping and quality control

*INSIG2* (rs7566605) genotyping was performed by the means of TaqMan allelic discrimination protocol (Applied Biosystems, Foster City, CA, USA). Genotype distributions confirmed Hardy-Weinberg equilibrium (HWE, *P*>0.05) in the non-obese healthy individuals. Genotype calling from real-time PCR data was performed using an algorithm called “best cycle genotyping algorithm”. The quality of the assignment of individual samples to clusters was determined on the basis of silhouette values[36].

### Statistical analysis

The independent samples *t*-test was used for two independent groups of obese and non-obese subjects to verify whether the means for two groups are significantly different or not. Genotype and allele distributions were compared between obese and non-obese subjects using Chi-squared test (X^2^). The independent segregation of alleles was tested for the HWE by comparing the observed genotype frequencies with the expected genotype frequencies (X^2^ test). For case-control studies, differences in genotype distributions were calculated using different models like log additive, recessive, and dominant model (adjusted for age and sex).

The differences among the three groups (genotypes) were assessed by one-way ANOVA for continuous variables, and variables were given as the mean±SD. The association of genotype of *INSIG2* variant (rs7566605) with obesity-associated phenotypes was performed with regard to different genetic models such as additive, dominant, and recessive model. Different models are used to ensure that the effect of genotype is due to the genetic variation or due to the other factors. ANOVA was used to analyze the correlation of obesity-associated phenotype between the genotypes. The statistical power of the study (>80%) was calculated by QUANTO 1.1 software with regards to the study type (case-control), disease prevalence (prevalence of obesity), and the frequency of the minor allele in the control population at the level of significance 0.05. All analyses of association between genotypes and phenotypes were conducted using SPSS (ver. 15), and *P*<0.05 was considered statistically significant.

## RESULTS AND DISCUSSION

Anthropometric, biochemical and clinical characteristics of the subjects are provided in Table 1. Fasting insulin, HOMA index, the percentage of body fat, fat mass, leptin, and adiponectin show significant difference between the groups, while fasting sugar did not differ between the obese and non-obese individuals.

**Table 1:** Clinical characteristics of study participants

Variable	Obese subjects* (n=309)	Non-obese subjects** (n=333)	*P* value
Age (Year)	36.78± 2.39	35.44± 2.15	0.916
Gender			
Male (%)	153 (49.5)	194 (58.3)	
Female (%)	156 (50.5)	139 (41.7)	
Weight (kg)	79.03±14.18	68.92±13.10	0.001
BMI (kg/m^2^)	33.49 ± 3.57	25.05 ± 3.37	<0.001
WC (cm)	102.30±12.39	95.67±11.05	<0.001
HC (cm)	105.38±11.09	100.83±9.17	<0.001
WHR (cm/cm)	0.97±0.10	0.95±0.08	0.015
SBP (mm Hg)	128.39±15.19	120.51±11.68	<0.001
DBP (mm Hg)	86.23±8.05	80.76±7.68	0.027
Insulin sensitivity			
F glucose (mg/dl)	109.23±15.94	109.64±18.62	0.065
F insulin (mU/ml)	14.99±9.73	10.27±6.01	<0.001
HOMA index	4.15±2.87	2.83±1.83	<0.001
Body fat (%)	37.28±6.166	27.86±6.12	<0.001
FM (kg)	30.60±8.33	20.60±8.16	<0.001
LPT (mg/ml)	21.13±4.34	14.73±6.59	<0.001
ADP (µg/mL)	6.52±1.88	7.81±1.61	<0.001
Lipid profile			
T cholesterol (mg/dl)	213.54±35.72	161.71±44.69	0.003
HDL-C (mg/dl)	42.82±7.13	46.30±10.16	0.001
Triglyceride (mg/dl)	130.28±28.88	107.12±19.57	0.008
LDL-C (mg/dl)	151.28±30.44	99.68±37.08	<0.001
VLDL-C (mg/dl)	26.06±5.78	25.03±4.14	0.008

Data were presented mean±SD. BMI, body mass index; WC, waist circumference; HC, hip circumference; WHR, waist-to-hip ratio; SBP, systolic blood pressure; DBP, diastolic blood pressure; F glucose, fasting glucose; F Insulin, fasting insulin; HOMA index, homeostasis model assessment index; FM, fat mass; LPT, leptin; ADP, adiponectin; T Cholesterol, total cholesterol; HDL-C, high density lipoprotein cholesterol; LDL-C, low density lipoprotein cholesterol; VLDL-C, very low density lipoprotein cholesterol.

* BMI≥30,

** BMI<30.

The SNP is polymorphic with minor allele frequencies of ≤27%, and the studied SNP followed HWE. The results of regression analysis are presented in Table 2. The analyzed genotypic data expressed that *INSIG2* (rs7566605) SNP was significantly associated with the increased risk of obesity (*P*≤0.001). C allele was significantly associated with the risk of obesity (*P*≤0.001). The *INSIG2* (rs7566605) showed an association with different obesity-associated phenotypes such as insulin, HOMA index, the percentage of body fat, fat mass, leptin, and adiponectin in both obese and non-obese subjects (Table 3).

**Table 2:** Genotype and allele frequency of *INSIG2* rs7566605 gene polymorphism in obese and non-obese subjects

*INSIG2* rs7566605 gene polymorphism	Obese subjects *n^a^* (*%*)	Non-obese subjects *n^a^* (*%*)	OR (95%CI)	*P* value
Genotype				
GG	139 (44.98)	197 (59.16)	Reference	Reference
GC	135 (43.69)	123 (36.94)	1.56 (1.12-2.16)	0.008
CC	35 (11.33)	13 (3.90)	3.82 (1.95-7.48)	<0.001
Allele				
G	413 (66.83)	517 (77.63)	Reference	Reference
C	205(33.17)	149 (22.37)	1.74 (1.36-2.22)	<0.001

a *n*=the number of individuals; Total number of obese (309) and non-obese subjects (333) (for genotype). Total number of chromosomes in obese (618) and non-obese subjects (666) (for alleles)

**Table 3:** Physiological parameters and genotypic classes for *INSIG2* rs7566605 gene polymorphism in obese and non-obese subjects

Variable	Obese subjects				Non-obese subjects			
	
	AA (n=139)	AG (n=135)	GG (n=35)	*P* value	AA (n=197)	AG (n=123)	GG (n=13)	*P* value
BMI	30.82±0.63	34.26±1.64	41.13±3.43	<0.001	22.91±2.69	27.98±0.95	29.82±0.08	<0.001
SBP (mm Hg)	126.98±11.19	128.79±12.72	125.66±11.65	0.267	119.55±10.25	121.55±12.27	120.77±8.78	0.284
DBP (mm Hg)	83.77±6.91	85.48±7.30	86.83±7.78	0.070	78.15±5.51	80.07±6.97	82.36±8.84	0.016
Insulin sensitivity								
F glucose (mg/dl)	110.48±21.19	108.58±15.75	110.38±18.22	0.675	107.64±15.07	111.84±16.84	108.55±17.94	0.071
F insulin (Mu/ml)	13.90±3.36	14.62±3.63	19.65±2.81	0.004	8.83±3.90	11.91±3.14	12.41±3.70	<0.001
HOMA Index	4.09±1.48	3.78±1.61	5.83±1.37	<0.001	2.39±1.45	3.40±1.73	3.49±1.05	<0.001
Body fat (%)	36.23±5.48	37.91±6.74	38.19±6.53	0.025	26.93±5.85	29.03±5.85	30.76±5.85	0.002
FM (kg)	28.85±6.54	31.16±7.89	32.25±9.68	0.002	18.95±6.75	22.84±9.55	24.46±7.61	<0.001
LPT (mg/ml)	19.22±5.45	22.59±8.80	23.06±5.88	<0.001	12.63±5.39	17.55±6.99	19.87±6.91	<0.001
ADP (µg/mL)	6.14±2.02	6.29±2.13	6.94±1.57	0.002	7.34±1.77	8.02±1.42	8.10±1.44	<0.001
Lipid Profile								
T Cholesterol (mg/dl)	156.64±35.71	167.28±42.48	150.64±29.68	0.094	147.48±20.37	148.55±25.93	141.55±18.00	0.560
HDL-C (mg/dl)	38.21±8.72	39.68±8.97	37.01±8.43	0.184	41.25±10.90	42.21±12.26	41.77±11.32	0.769
Triglyceride (mg/dl)	113.30±19.77	116.83±21.95	112.07±19.06	0.269	105.07±17.53	105.29±18.52	110.82±18.49	0.534
LDL-C (mg/dl)	118.49±27.18	124.21±30.88	113.59±23.84	0.083	106.17±22.45	107.91±27.39	111.80±12.07	0.631360
VLDL-C (mg/dl)	22.66±3.95	23.37±4.39	22.41±3.81	0.269	21.01±3.51	21.06±3.70	22.16±3.70	0.534

Data were presented mean±SD. BMI, body mass index; SBP, systolic blood pressure; DBP, diastolic blood pressure; F glucose, fasting glucose; F Insulin, fasting insulin; HOMA Index, homeostasis model assessment index; FM, fat mass; LPT, leptin; ADP, adiponectin; T Cholesterol, total cholesterol; HDL-C, high density lipoprotein cholesterol; LDL-C, low density lipoprotein cholesterol; VLDL-C, very low density lipoprotein cholesterol.

The role of *INSIG2* variant (rs7566605) as a determining factor for development of obesity was investigated in a sample of 642 obese and non-obese individuals belonging to the north of India. In contrast to Kumar *et al*.[17], a significant association was found between *INSIG2* variant (rs7566605) with obesity. Herbert *et al*.[9] also reported a significant association between *INSIG2* genetic variant (rs7566605) and obesity, as assessed by a BMI≥30 kg/m^2^ in different populations, including Western European ancestry, African-Americans, and children. However, in the Nurses’ Health Study cohort, the absence of such association suggested that the SNP may have variable effects on different populations.

The association between *INSIG2* genetic variant (rs7566605) and obesity has been reported in some studies[12,13,37], but this association was not replicated in others[11,15,16]. However, the association was controversial in Asian populations[27,38], except for Japanese population[18]. Most importantly, no relationship was observed between rs7566605 and BMI or the measures of obesity in two separate studies, using different cohorts of Indian population[17,18].

Minor allele frequency was around 0.27% in the present study, which is comparable to the frequency reported in a previous study on Indian population[17]. The CC genotype and C allele were significantly associated with obesity in north India, as reported before[9,11-13]. In contrast to a previous study on Indian population[17], we observed a significant association with obesity. This discrepancy may be that Kumar *et al*.[17] used two different cohorts, one with three linguistic lineages (Indo-European, Dravidian, and Tibeto-Burman) and the other with coronary artery disease cases and controls. Smith *et al*.[18], recruited type two diabetic case and controls. In contrast to these studies, the present study has been done on non-diseased individuals of north Indian origin.

On account of the genetic diversity in different populations, the extent of linkage disequilibrium among the genetic variants is likely to vary, which may be one of the most important reasons for the reported inconsistent findings. In the present study, we selected only individuals of the North Indian origin, and all of them were free from any kind of disease.

The influence of *INSIG2* rs7566605 on variation in other anthropometric measures, including systolic blood pressure, diastolic blood pressure, fasting insulin, HOMA index, the percentage of body fat, fat mass, leptin, adiponectin, and lipid profile were analyzed in this study. We observed significant association with fasting insulin, HOMA index, percentage of body fat, and fat mass. Significant mean differences were also observed among *INSIG2* rs7566605 genotypes for leptin and adiponectin in obese and non-obese study participants. According to the Cervino *et al*.[23], *INSIG2* might be associated with fat mass, as INSIG2 was recognized the upstream of several obesity-related genes in the transcriptional network. Another polymorphism, *INSIG2* -102G/A, in high linkage disequilibrium with rs7566605, is associated with obesity and with adipogenesis through SREBP1 activation[26,39]. The -102G/A polymorphism has been anticipated as the functional polymorphism of *INSIG2* since it seems to influence the level of *INSIG2* expression. Dipple *et al*.[40] suggested that C alleles of *INSIG2* likely influence fat metabolism in relation to physical activity, accommodating the complication within biological networks, and genotype–phenotype correlation. Orkunoglu-Suer and co-workers[37] reported that the rs7566605 C allele is significantly associated with increased fat in female subjects. This elevation may be related to greater insulin sensitivity, which mediates through an increase in free fatty acid synthase and may also alter with *INSIG2* genotypes. The finding has particularly interesting in regard to our results for the associations of rs7566605 genotypes with fat and insulin.

The *INSIG2* has a role in the regulation of fat metabolism and insulin resistance[41,42]. Meanwhile, rs7566605 SNP presents approximately 10-kb upstream from *INSIG2*; therefore, it may affect the transcriptional activity of *INSIG2*. *INSIG2* is expressed ubiquitously. It was down-regulated by insulin in the liver and involved in fatty acid synthesis[22,43].

It has previously been shown that plasma adiponectin level could be influenced by body fat[30]. In the present study, plasma adiponectin levels differ significantly among the SNP rs7566605 genotypes in obese and non-obese participants, which indicates the genetic effect of this polymorphism not only on obesity but also on body fat. The observed associations with fasting plasma insulin and HOMA index further supports this possibility. In a previous animal study, adiponectin-knockout mice on a high-salt diet developed obesity-associated phenotype like hypertension, which was ameliorated by adiponectin intervention[44]. Adiponectin reduced circulating fatty acid levels via enhanced fatty acid oxidation and reduced fatty acid synthesis[45], so it may associate with the increased risk of obesity-associated phenotype, most especially, hypertension through its adverse effects on fatty acid metabolism.

Leptin is an endocrine hormone that inhibits food intake and increases energy expenditure by acting on the hypothalamus[46]. In the present study, the SNP rs7566605 genotypes were significantly associated with plasma leptin levels. Leptin plays a major role in the body fat storage through the regulation of food intake and total body energy consumption, and its circulating levels correlate closely with both the BMI and the total amount of body fat[47]. *INSIG2* also intermediates feedback control of cholesterol synthesis[21], though serum total cholesterol, HDL-C, triglycerides, LDL-C, and very low density lipoprotein cholesterol were not significantly different among genotypes, but it is possible that *INSIG2* is associated with obesity as it affects lipid metabolism.

In summary, our study indicates that rs7566605 in the upstream region of the *INSIG2* gene may influence the risk of obesity and the development of obesity- associated phenotypes. Therefore, the genetic effects of the *INSIG2* gene on obesity and obesity-associated phenotypes such as fat mass, insulin, adiponectin, and lipids can be clarified by further studies with the new putative functional variant. Further, these effects may strengthen our understanding toward the role of this gene in susceptibility to obesity and obesity-associated phenotypes, which may help to explain metabolic and cardiovascular dysfunction.
